# Comparison of plasma neurofilament light and total tau as neurodegeneration markers: associations with cognitive and neuroimaging outcomes

**DOI:** 10.1186/s13195-021-00944-y

**Published:** 2021-12-14

**Authors:** Jordan D. Marks, Jeremy A. Syrjanen, Jonathan Graff-Radford, Ronald C. Petersen, Mary M. Machulda, Michelle R. Campbell, Alicia Algeciras-Schimnich, Val Lowe, David S. Knopman, Clifford R. Jack, Prashanthi Vemuri, Michelle M. Mielke, for the Alzheimer’s Disease Neuroimaging Initiative

**Affiliations:** 1 Medical Scientist Training Program, Mayo Clinic Alix School of Medicine, Rochester, MN USA; 2 Department of Quantitative Health Sciences, Mayo Clinic, Rochester, MN USA; 3 Department of Neurology, Mayo Clinic, Rochester, MN USA; 4 Department of Psychiatry and Psychology, Mayo Clinic, Rochester, MN USA; 5 Department of Laboratory Medicine and Pathology, Mayo Clinic, Rochester, MN USA; 6 Department of Radiology, Mayo Clinic, Rochester, MN USA

**Keywords:** Neurofilament light chain, Total tau, Blood-based biomarker, Cognition, Neuroimaging

## Abstract

**Background:**

Total tau protein (T-Tau) and neurofilament light chain (NfL) have emerged as candidate plasma biomarkers of neurodegeneration, but studies have not compared how these biomarkers cross-sectionally or longitudinally associate with cognitive and neuroimaging measures. We therefore compared plasma T-Tau and NfL as cross-sectional and longitudinal markers of (1) global and domain-specific cognitive decline and (2) neuroimaging markers of cortical thickness, hippocampal volume, white matter integrity, and white matter hyperintensity volume.

**Methods:**

We included 995 participants without dementia who were enrolled in the Mayo Clinic Study of Aging cohort. All had concurrent plasma NfL and T-tau, cognitive status, and neuroimaging data. Follow-up was repeated approximately every 15 months for a median of 6.2 years. Plasma NfL and T-tau were measured on the Simoa-HD1 Platform. Linear mixed effects models adjusted for age, sex, and education examined associations between baseline *z*-scored plasma NfL or T-tau and cognitive or neuroimaging outcomes. Analyses were replicated in Alzheimer’s Disease Neuroimaging Initiative (ADNI) among 387 participants without dementia followed for a median of 3.0 years.

**Results:**

At baseline, plasma NfL was more strongly associated with all cognitive and neuroimaging outcomes. The combination of having both elevated NfL and T-tau at baseline, compared to elevated levels of either alone, was more strongly associated at cross-section with worse global cognition and memory, and with neuroimaging measures including temporal cortex thickness and increased number of infarcts. In longitudinal analyses, baseline plasma T-tau did not add to the prognostic value of baseline plasma NfL. Results using ADNI data were similar.

**Conclusions:**

Our results indicate plasma NfL had better utility as a prognostic marker of cognitive decline and neuroimaging changes. Plasma T-tau added cross-sectional value to NfL in specific contexts.

**Trial registration:**

Not applicable

## Introduction

There are several potential markers of neurodegeneration that can aid in capturing a range of brain changes and pathologies. Plasma biomarkers are advantageous over CSF and imaging markers in that they provide a low-cost, non-invasive option of screening for neurodegeneration and for assessing rate of disease progression given the feasibility of repeat blood draws. It is important to understand what information each plasma marker of neurodegeneration provides to inform how they can best be utilized for clinical and research purposes.

Neurofilament light chain (NfL) and total tau (T-tau) protein have been examined as candidate blood-based biomarkers of neurodegeneration [1]. Multiple studies have shown, cross-sectionally and longitudinally, across neurodegenerative diseases, that elevated levels of plasma NfL and T-tau are associated with worse cognition and neuroimaging measures of cortical thickness, cortical atrophy, white matter hyperintensity (WMH), or white matter integrity [2–14]. Recent studies have compared plasma NfL and T-tau as prognostic markers of cognitive outcomes in patients with mild dementia [15], and of cognitive decline and risk of dementia among people without dementia [16]. However, studies have not compared how these two plasma markers cross-sectionally or longitudinally associate with neuroimaging changes and vascular pathology in the years preceding dementia. In the current study, we compared associations of plasma NfL and T-tau among individuals without dementia as cross-sectional and longitudinal markers of global and domain-specific cognitive decline, and with neuroimaging markers of cortical thickness, hippocampal volume, white matter integrity, and WMH volume in the community-based Mayo Clinic Study of Aging (MCSA). We also determined whether the combination of elevated levels of both plasma NfL and T-tau at baseline were more strongly associated cross-sectionally and longitudinally with each outcome compared to each plasma marker alone. We further examined how these relationships were altered by the presence of amyloid-beta (Aβ) pathology. Lastly, the MCSA is population-based and the only exclusionary criteria are individuals who are terminally ill or in hospice. Participants are not excluded based on history of cerebrovascular disease or recent psychiatric conditions. Plasma levels of both NfL and T-tau have been found to be elevated in stroke patients, and among those with other cardiovascular conditions [2, 17]. Therefore, we also compared the utility of plasma T-tau and NfL in the Alzheimer’s Disease Neuroimaging Initiative (ADNI), which excludes a subset of participants with these conditions [18], to determine whether associations differed by study population.

## Methods

### Mayo Clinic Study of Aging

The MCSA is a prospective population-based study examining the epidemiology of cognitive decline and risk of mild cognitive impairment (MCI) among residents living in Olmsted County, Minnesota [19]. In 2004, Olmsted County residents between the ages of 70 and 89 were enumerated using the Rochester Epidemiology Project medical records-linkage system in an age- and sex-stratified random sampling design [20]. The study was extended to include those aged 50 and older in 2012. The present study consists of 995 MCSA non-demented participants with measures of both plasma NfL and T-tau.

### Standard protocol approvals, registrations, and patient consents

The study was approved by Mayo Clinic and Olmsted Medical Center Institutional Review Boards. Written informed consent was obtained from all participants.

MCSA visits include an interview by a study coordinator, physician examination, and neuropsychological testing, as previously published [19]. Clinical follow-up visits occur at 15-month intervals. Neuropsychological testing included nine tests covering four domains: memory [Auditory Verbal Learning Test Delayed Recall Trial [21], Wechsler Memory Scale-Revised Logical Memory-II and Visual Reproduction-II [22], language [Boston Naming Test [23] and category fluency] [24], visuospatial skills [WAIS-R Picture Completion and Block Design subtests] [25], and attention [Trailmaking Test B [24, 26] and WAIS-R Digit Symbol subtest [25]]. Using the mean and standard deviation (SD) from baseline, test scores were converted to *z*-scores and *z*-scores within each domain were averaged and *z*-scored for a domain-specific *z*-score. Global cognition was calculated using the *z*-transformed average of the four cognitive domain *z*-scores.

### MCI and dementia diagnostic determination

Clinical diagnoses were determined by a consensus committee. Cognitive performance was compared with the age-adjusted scores of individuals previously obtained in a separate sample using Mayo’s Older American Normative Studies [27]. Participants with scores around 1.0 SD below the age-specific mean in the general population were considered for possible cognitive impairment. The operational definition of MCI was based on clinical judgment including a history from the patient and informant and cognitive performance. Published criteria were used for the diagnosis: cognitive complaint, cognitive function not normal for age, essentially normal functional activities, and no dementia [28]. A final diagnosis was made after considering education, occupation, and visual or hearing deficits and reviewing all other participant information. The diagnosis of dementia was based on DSM-IV criteria [29]. Participants who performed in the normal range and did not meet criteria for MCI or dementia were deemed cognitively unimpaired (CU).

### Structural MRI

Neuroimaging occurred at 15- or 30-month intervals. Structural magnetic resonance imaging (MRI) was acquired using standardized Magnetization Prepared – Rapid Gradient Echo (MPRAGE) sequences on 3T GE scanners (GE Medical Systems, Milwaukee, WI) as well as Siemens scanners. Hippocampal volume and cortical thickness were measured with FreeSurfer (version 5.3). Each participant’s raw hippocampal volume was adjusted for total intracranial volume (TIV) to create a TIV-adjusted hippocampal volume [30]. We used lobar cortical thickness measures for temporal (entorhinal, parahippocampal, banks of superior temporal sulcus, fusiform, inferior temporal, insula, middle temporal, superior temporal, temporal pole, transverse temporal); frontal (caudal middle frontal, frontal pole, lateral orbitofrontal, medial orbitofrontal, pars opercularis, pars orbitalis, pars triangularis, rostral middle frontal, superior frontal); parietal (inferior parietal, postcentral, precuneus, superior parietal, supramarginal); and occipital (cuneus, lateral occipital, lingual, pericalcarine) lobes.

Diffusion tensor imaging (DTI) sequences were processed and analyzed for fractional anisotropy (FA) of the corpus callosum as previously described [31, 32]. Loss of white matter microstructural integrity measured using DTI has been shown to be a good indicator of axonal injury. We used the JHU atlas to regionally measure FA from DTI scans [33].

White matter hyperintensities on standard 2-dimensional fluid-attenuated inversion recovery imaging were segmented and edited by a trained imaging analyst using a semi-automated method, as previously described [34, 35]. WMH volume is presented as the percentage of TIV.

### Amyloid PET imaging

Aβ PiB-PET images were acquired using a PET/CT scanner (DRX, GE Healthcare) operating in 3-dimensional mode [36]. Pittsburgh compound B (PiB)–PET scan, consisting of 4 5-min dynamic frames, was acquired from 40 to 60 min after injection [37, 38]. Quantitative image analysis for PiB was done using our in-house fully automated image processing pipeline [39]. A global cortical PiB-PET retention ratio was computed by calculating the median uptake over voxels in the prefrontal, orbitofrontal, parietal, temporal, anterior cingulate, and posterior cingulate/precuneus regions of interest for each participant and dividing this by the median uptake over voxels in the cerebellar crus. No partial volume correction was used. The atlas and image recognition steps were based on a 3D T1-weighted volume MRI sequence. We dichotomized participants as having elevated brain amyloid based on a cutoff of 1.48 standard uptake value ratio (SUVR) [31].

### Blood collection and plasma assays

Participants’ blood was collected in clinic after an overnight fast. The blood was centrifuged, aliquoted, and stored at −80°C. Briefly, after thawing and mixing, plasma samples were centrifuged 5 min × 10,000*g*. Plasma T-tau and NfL were measured on the Quanterix HD-1 analyzer using the Simoa® Neurology 3-Plex A (N3PA) (catalog #101995) and the Simoa® NF-light (catalog #103186) Advantage kits per the manufacturer’s instructions. Samples were diluted 1:4 using the instrument’s onboard dilution protocol and run in singlet. Eight-point calibration curves and sample measurements were determined on Simoa® HD-1 Analyzer software using a weighting factor 1/Y^2^ and a 4-parameter logistic curve fitting algorithm. Two levels of quality control material included in respective kits were included in runs, flanking the samples at the front and end of each batch. In the N3PA kits, plasma tau is quantified using a capture antibody that binds to the proline-rich P2 region in the mid-domain of the tau protein. The detection antibody binds at the N-terminal of the tau protein in which tyrosine 18 is not phosphorylated. The tau calibration curve was generated using a recombinant human tau 381 isoform with a single N-terminal insert and three microtubule binding domain repeats (3R/1N). In the NF-light kits, both the capture and detector antibodies (Uman Diagnostics article #27016-100, #27017-100) bind to the conserved rod domain of the NfL protein. Internal studies of imprecision for T-tau and NfL, respectively, are as follows: intra-assay imprecision (approximate concentrations of 2.00 and 75.0 pg/mL and 7.00 and 72.0 pg/mL) was 5.9% and 3.3% and 5.6% and 2.0%. Inter-assay precision (approximate concentrations of 2.00 and 75.0 pg/mL and 5.00 and 150.0 pg/mL) was 7.0% and 6.5% and 17.0% and 5.6%.

### ADNI methods

ADNI Study Design Data were obtained from the database (http://adni.loni.usc.edu). The ADNI was launched in 2003 as a public-private partnership, led by principal investigator Michael W. Weiner, MD (the most recent information on the ADNI is available at http://www.adni-info.org). The ADNI participants have been recruited from more than 50 sites across the USA and Canada. For the present study, we used data obtained from the Laboratory of Neuroimaging (University of Southern California) ADNI database on September 11, 2020. The study data and samples were collected from September 7, 2005, to February 13, 2012. Regional ethical committees of all participating institutions approved the ADNI. All study participants provided written informed consent.

### ADNI participants

The current ADNI analysis consisted of all CU and MCI participants with available baseline plasma NFL and T-tau samples from ADNI-1. Inclusion and exclusion criteria were described in detail previously [40]. Briefly, all ADNI-1 participants were aged 55 to 90 years, had completed at least 6 years of education, were fluent in Spanish or English, and had no substantial neurological disease other than Alzheimer’s disease (AD). Controls had Mini-Mental State Examination (MMSE) [41] scores of 24 or higher, and a Clinical Dementia Rating (CDR) [42] score of 0. Patients with MCI had MMSE scores of 24 or higher, objective memory loss tested by delayed recall of the Wechsler Memory Scale logical memory II (adjusted for education), a CDR score of 0.5, preserved activities of daily living, and absence of dementia. The ADNI cognitive outcomes examined included *z*-scored results of the Alzheimer’s Disease Assessment Scale-Cognitive 13 (ADAS-COG 13) [18], and *z* log-transformed results of the Trail Making Test Part B, both for which higher scores indicate worse performance. Cognitive outcomes also included *z*-transformed immediate and delayed logical memory, in which higher scores indicated better performance. Structural brain images were acquired with 1.5T MRI scanners with T1-weighted MRI scans using a sagittal volumetric magnetization prepared rapid gradient echo sequence. FreeSurfer was used to quantitate hippocampal volume, which was adjusted for total intracranial volume [43]. Plasma NfL and T-tau were measured on the Quanterix HD-1 analyzer. Plasma NfL concentrations were measured using a NfL kit (NF-light; UmanDiagnostics), transferred to the ultrasensitive single-molecular array platform using a home brew kit (Simoa Homebrew Assays Development Kit; Quanterix Corporation). Plasma tau was analyzed with the Human Total Tau kit (Quanterix, Lexington, MA) using a monoclonal capture antibody that reacts with a linear epitope in the midregion of all tau isoforms and a detection antibody that reacts with a linear epitope in the N-terminal region of T-tau.

### Statistical analysis

Wilcoxon rank-sum tests were used to examine differences in plasma NfL and T-tau levels by clinical diagnosis (CU vs. MCI). For all models comparing the cross-sectional or longitudinal utility of the plasma markers, plasma NfL and total tau were z-scored to directly compare the coefficients. Linear mixed effects models were used for both the MCSA and ADNI cohorts to examine baseline continuous measures of plasma T-tau and NfL in relation to both cross-sectional and longitudinal cognitive and neuroimaging outcomes. In addition, we assessed whether participants having both plasma T-tau and NfL in the top quartile, compared to either alone, was more strongly associated with cross-sectional and longitudinal cognitive and imaging outcomes. All linear mixed effects models used random participant-specific intercepts and slopes for time. Multivariable models adjusted for age, sex, education, and previous exposure to the cognitive battery. We summarized the models in tables using beta coefficients, 95% confidence intervals (CI), and *p*-values. To visualize the results, corresponding forest plots have been created, which plot the beta coefficients and 95% CIs. The usual alpha level of 0.05 was utilized to determine statistical significance. All analyses were completed using SAS version 9.4 (SAS Institute, Cary, NC) and R version 3.6.2 (R Foundation for Statistical Computing, Vienna, Austria).

## Results

Demographic and clinical data, as well as baseline cognitive and imaging data for the MCSA cohort, are summarized in Table 1 by cognitive status. Of the 995 participants, 864 were CU and 131 had MCI. The median (interquartile range [IQR]) age of all individuals was 76.3 (68.1, 81.8) years and 56.1% were male. MCI participants, compared to CU, had higher levels of both plasma NfL (23.1 [16.7, 32.5] vs. 17.0 [11.8, 23.9] pg/mL, *p* < 0.001) and T-tau (2.83 [1.94, 3.59] vs. 2.53 [1.82, 3.30] pg/mL, *p* = 0.039). Median follow-up (IQR) after the first plasma measurement was 6.2 (4.2, 7.4) years. Median total number of infarcts in the cohort was 0 (range 0–8).

**Table 1:** Mayo Clinic Study of Aging participant baseline characteristics

Characteristic	Data available	CU	Data available	MCI	***p*** value
		Median (IQR) / ***N*** (%)		Median (IQR) / ***N*** (%)	
Age (years)	864	75.6 (67.0, 80.9)	131	81.4 (75.6, 85.3)	<0.0001^a^
Male	864	479 (55.4%)	131	79 (60.3%)	0.2957^b^
Education (years)	864	15.0 (12.0, 16.0)	131	13.0 (12.0, 15.0)	<0.0001^a^
≥ 1 APOE ε4	864	228 (26.4%)	131	45 (34.4%)	0.0570^b^
Plasma total tau (pg/mL)	864	2.53 (1.82, 3.30)	131	2.83 (1.94, 3.60)	0.0391^a^
Plasma NfL (pg/mL)	864	17.0 (11.8, 23.9)	131	23.1 (16.7, 32.5)	<0.0001^a^
Body mass index (kg/m^2^)	860	27.5 (24.9, 31.0)	130	26.9 (23.6, 29.7)	0.0211^a^
Hypertension	864	571 (66.1%)	131	100 (76.3%)	0.0197^b^
Stroke	864	21 (2.4%)	131	10 (7.6%)	0.0014^b^
Myocardial infarction	864	96 (11.1%)	131	29 (22.1%)	0.0004^b^
Follow-up time (years)	864	6.2 (4.9, 7.5)	131	5.0 (2.7, 6.3)	<0.0001^a^
Cognitive *z*-score					
Global	826	0.242 (−0.338, 0.830)	122	−1.325 (−1.953, −0.984)	<0.0001^a^
Memory	859	0.259 (−0.386, 0.835)	128	−1.470 (−1.902, −0.809)	<0.0001^a^
Language	844	0.243 (−0.400, 0.777)	126	−1.067 (−1.646, −0.390)	<0.0001^a^
Visuospatial ability	841	0.216 (−0.346, 0.761)	124	−1.140 (−2.068, −0.370)	<0.0001^a^
Attention	841	0.181 (−0.444, 0.761)	125	−0.765 (−1.565, −0.061)	<0.0001^a^
Cortical thickness					
Frontal	583	2.325 (2.224, 2.411)	85	2.236 (2.160, 2.337)	<0.0001^a^
Parietal	582	2.629 (2.540, 2.729)	85	2.504 (2.406, 2.610)	<0.0001^a^
Temporal	583	2.076 (1.987, 2.169)	85	1.987 (1.924, 2.076)	<0.0001^a^
Occipital	583	1.879 (1.800, 1.958)	85	1.808 (1.753, 1.886)	<0.0001^a^
Amyloid PET ≥ 1.48 SUVR	441	129 (29.3%)	59	39 (66.1%)	<0.0001^a^
Hippocampal volume (cm^3^)	584	7.35 (6.84, 7.89)	85	6.85 (6.22, 7.21)	<0.0001^a^
WMH volume (mm^3^)	341	0.005 (0.003, 0.011)	53	0.014 (0.008, 0.021)	<0.0001^a^
Corpus callosum FA	369	0.643 (0.614, 0.662)	36	0.625 (0.565, 0.648)	0.0036^a^
Infarcts (total)	419	0 (0)	48	0 (0, 1)	<0.0001^a^

Hippocampal volume measures were adjusted for total intracranial volume

*APOE* apolipoprotein E, *FA* fractional anisotropy, *IQR* interquartile range, *MCI* mild cognitive impairment, *NfL* neurofilament light, *WMH* white matter hyperintensity

^a^Kruskal Wallis rank sum test

^b^Pearson’s Chi-Square test

### Baseline plasma NfL and T-tau and global and domain-specific cognitive z-scores

**Fig. 1:**
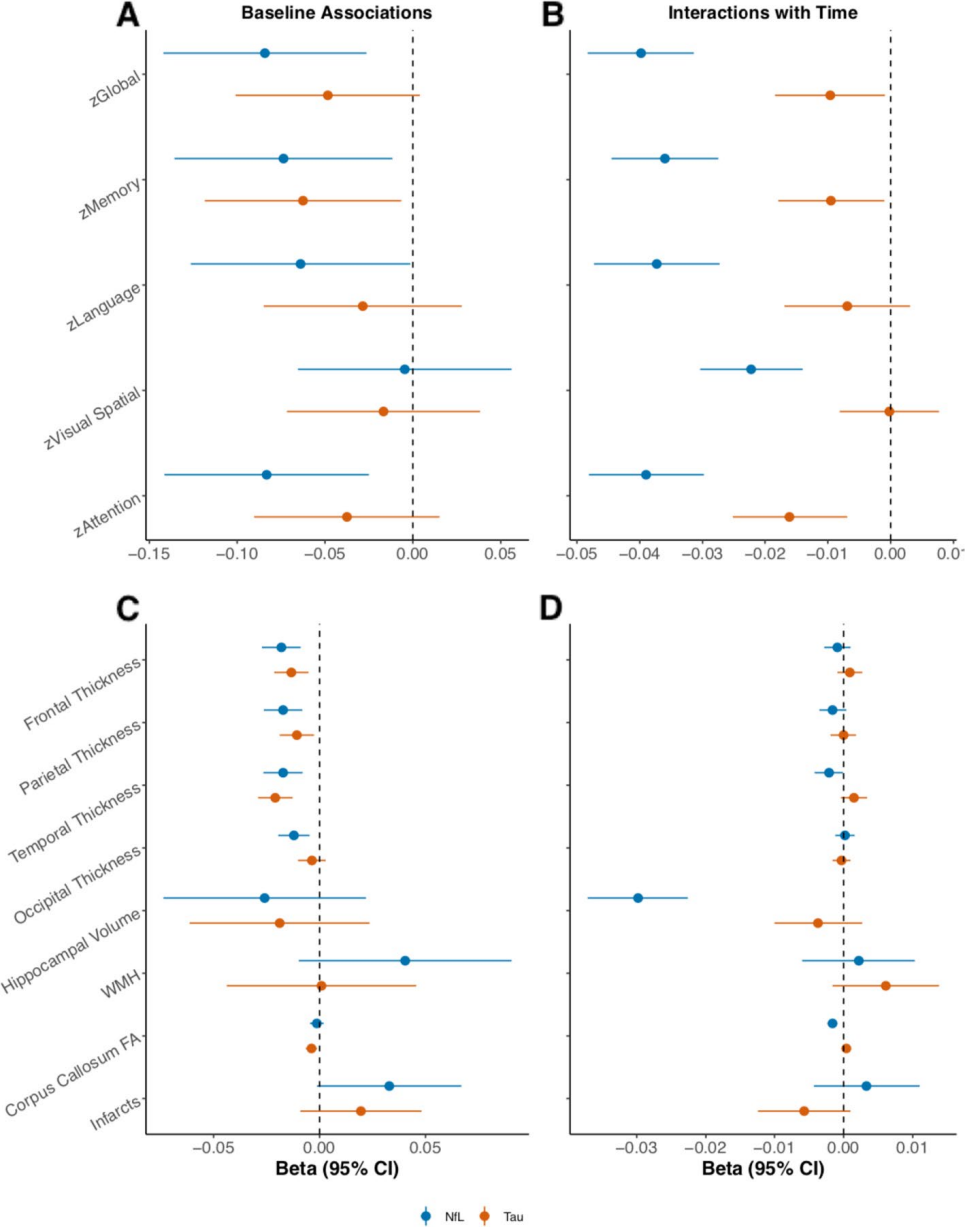
Relationships between plasma NfL or T-tau and cognition and neuroimaging in the MCSA. **A** Associations of baseline plasma NfL or T-tau with cognitive *z*-scores at cross-section. **B** Associations of baseline plasma NfL or T-tau with longitudinal cognitive outcomes. **C** Associations of baseline plasma NfL or T-tau with cross-sectional neuroimaging measures. **D** Associations of baseline plasma NfL or T-tau and longitudinal neuroimaging changes. Models adjust for age, sex, education, and whether or not the cognitive test had been previously administered. WMH volume and infarct measurements were log-transformed. Hippocampal volume measures were adjusted for total intracranial volume. Median follow-up was 6.2 years. *Abbreviations: FA* fractional anisotropy, *NfL* neurofilament light, *T-tau* total tau, *WMH* white matter hyperintensity

Figure 1 illustrates associations between *z*-transformed baseline plasma NfL or T-tau and global and domain-specific cognitive *z*-score. Cross-sectionally (Fig. 1A), each one SD increase in plasma NfL at baseline was associated with worse scores in domains of memory, language, and attention, and with global cognition. Higher baseline plasma T-tau was only associated with worse performance in memory.

Longitudinally, elevated baseline plasma NfL was associated with declines in global cognition and with all domain-specific *z*-scores (all *P* < 0.001; Fig. 1B). In contrast, higher baseline plasma T-tau was only associated with declines in memory, attention, and global cognitive *z*-scores. Among the domains in which both high plasma NfL and T-tau were associated with cognitive decline, the association was always stronger for NfL.

### Examination of the combination of plasma NfL and T-tau for cognitive decline

We next examined whether the combination of both elevated NfL and T-tau levels was more strongly associated with worse global and domain-specific cognition than either alone (Table 2). Cross-sectionally, having plasma NfL in the top quartile (Q4) was associated with worse attention. There was no cross-sectional relationship between Q4 plasma T-tau and any cognitive measure. The combination of having both plasma NfL and T-tau in Q4, compared to all in the bottom three quartiles, was associated with lower memory, attention, and global *z*-scores. However, T-tau did not provide added information beyond NfL in relation to attention *z*-score. Thus, the combination of elevated plasma NfL and T-tau was more strongly associated with memory and global cognition than either alone.

**Table 2:** Cross-sectional and longitudinal cognition in those with baseline plasma NfL, T-tau, or both in the top quartile

Cognitive measure (***z-***score)	Beta	Lower CI	Upper CI	***p*** value	Beta	Lower CI	Upper CI	***p*** value
	**zNfL Q4**				zNfL Q4 * Time			
Memory	0.002	−0.169	0.174	0.979	−0.083	−0.106	−0.060	< 0.001
Language	−0.132	−0.305	0.041	0.134	−0.086	−0.114	−0.059	< 0.001
Attention	−0.208	−0.369	−0.047	0.012	−0.093	−0.118	−0.068	< 0.001
Visuospatial	−0.067	−0.235	0.102	0.439	−0.065	−0.087	−0.043	< 0.001
Global	−0.134	−0.295	0.028	0.105	−0.102	−0.126	−0.078	< 0.001
	**zT-tau Q4**				zT-tau Q4 * Time			
Memory	−0.015	−0.174	0.143	0.851	−0.008	−0.030	0.014	0.453
Language	−0.086	−0.246	0.074	0.292	−0.014	−0.040	0.012	0.283
Attention	−0.061	−0.209	0.089	0.427	−0.010	−0.033	0.014	0.408
Visuospatial	−0.021	−0.177	0.134	0.787	−0.002	−0.022	0.019	0.865
Global	−0.053	−0.202	0.095	0.481	−0.014	−0.036	0.009	0.240
	**Q4 Both**				Q4 Both * Time			
Memory	−0.261	−0.462	−0.059	0.011	−0.071	−0.099	−0.043	< 0.001
Language	−0.146	−0.350	0.058	0.162	−0.077	−0.110	−0.044	< 0.001
Attention	−0.221	−0.410	−0.031	0.023	−0.094	−0.124	−0.063	< 0.001
Visuospatial	−0.104	−0.302	0.094	0.305	−0.042	−0.069	−0.016	0.002
Global	−0.260	−0.449	−0.070	0.008	−0.090	−0.119	−0.061	< 0.001

The reference group is having both biomarkers in the bottom three quartiles. Models adjust for age, sex, education, and whether or not the cognitive test had been previously administered

*CI* confidence interval, *NfL* neurofilament light, *Q4* top quartile, *T-tau* total tau

Longitudinally, baseline Q4 plasma NfL, compared to the bottom three quartiles, was associated with declines in all global and domain-specific *z*-scores (Table 2). In contrast, baseline Q4 T-tau was not associated with decline in any cognitive domain. The combination of both Q4 NfL and T-tau was associated with declines in all global and domain-specific *z*-scores, but these associations were driven by NfL. Elevated plasma T-tau therefore did not contribute to the prognosis of cognitive decline.

### Baseline plasma NfL and T-tau and neuroimaging measures

The cross-sectional associations between *z*-transformed baseline plasma NfL or T-tau and neuroimaging measures are shown in Fig. 1C. Each one SD increase in plasma NfL was associated with lower cortical thickness in all lobar regions. Elevated plasma T-tau was also associated with lower thickness of all cortical regions except occipital thickness. In addition, each one SD increase in plasma T-tau was associated with decreased FA of the corpus callosum.

Longitudinally (Fig. 1D), higher baseline plasma NfL was associated with declines in temporal cortex thickness, corpus callosum FA and hippocampal volume. Baseline plasma T-tau was not associated with change in any neuroimaging outcomes.

### Examination of the combination of plasma NfL and T-tau for neuroimaging outcomes

We also examined whether the combination of plasma NfL and T-tau in Q4, compared to one in the top quartile or both in the bottom three quartiles, was more strongly associated with neuroimaging outcomes than either alone (Table 3). Cross-sectionally, Q4 NfL alone was not associated with any neuroimaging outcome. Participants with only plasma T-tau in Q4 had decreased thickness of temporal cortex. In contrast, the combination of having both plasma NfL and T-tau in the Q4 was associated with lower temporal thickness and a higher number of infarcts. Notably, these associations were stronger than those between Q4 T-tau alone and neuroimaging, suggesting that the combination of elevated T-tau and NfL provided more information than either alone in cross-sectional assessments of these neuroimaging outcomes.

**Table 3:**
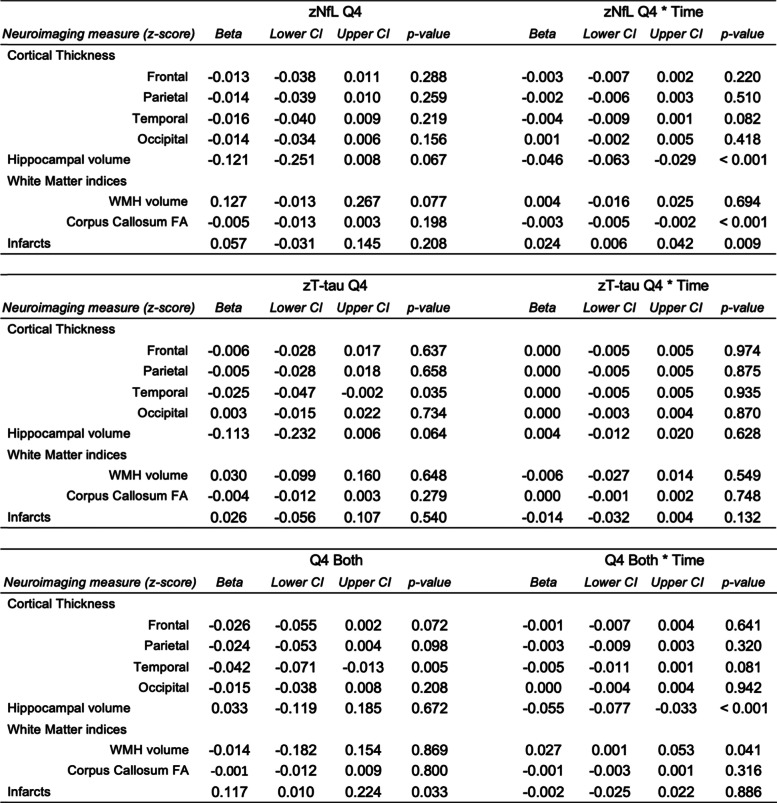
Cross-sectional and longitudinal neuroimaging associations for baseline plasma NfL, T-tau, or both in the top quartile

Reference level is having both biomarkers in the bottom three quartiles. Models adjust for age, sex, and education. WMH volume and infarct measurements were log-transformed. Hippocampal volume measures were adjusted for total intracranial volume

*CI* confidence interval, *FA* fractional anisotropy, *NfL* neurofilament light, *Q4* fourth quartile, *T-tau* total tau, *WMH* white matter hyperintensity

Longitudinally, plasma NfL in Q4 was associated with decline in corpus callosum FA and hippocampal volume. Q4 T-tau alone was not associated with change in any neuroimaging outcome. The combination of both NfL and T-tau in Q4 was associated with increasing WMH volume and declines in hippocampal volume. Thus, the combined information provided by both markers better predicted an increase in WMH volume compared to either alone.

### Cognitive and imaging associations of plasma NfL and T-tau by elevated brain amyloid

To examine whether elevated brain amyloid influenced the associations of plasma NfL and T-tau with cognitive or neuroimaging outcomes, we also ran models including an interaction with elevated baseline brain amyloid, defined by having an amyloid PET ≥ 1.48 SUVR. Of the 995 participants, 500 (50.3%) had amyloid PET data. Of the 441 CU individuals with amyloid PET data, 129 (29.3%) had elevated brain amyloid, while 39 of the 59 individuals with MCI (66.1%) had elevated brain amyloid (Table 1). Those with an amyloid PET scan had lower median NfL than those without a scan (16.7 vs. 19.0, *p* < 0.001), but no differences were seen for T-tau.

**Fig. 2:**
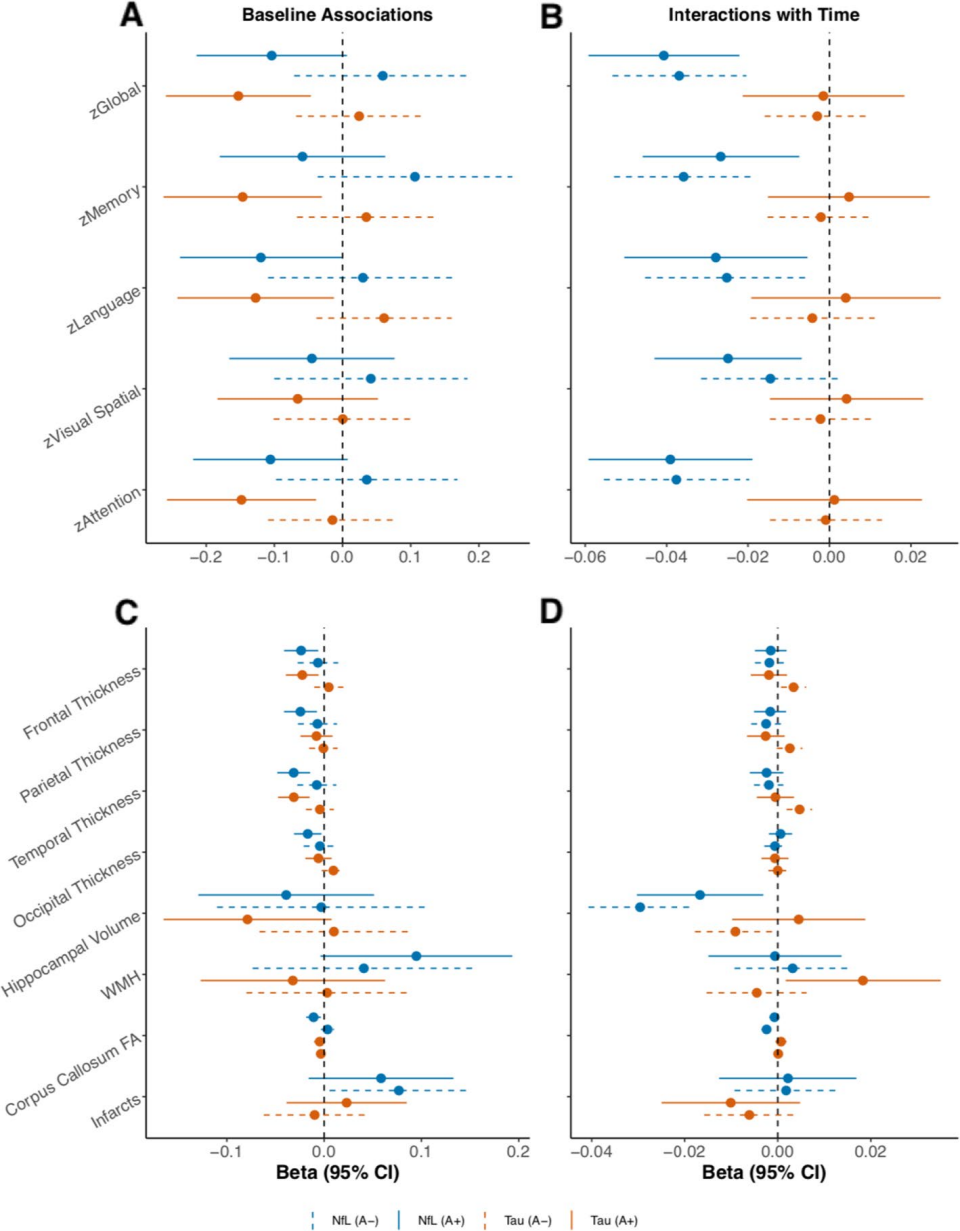
Amyloid-dependent associations between biomarkers with cross-sectional and longitudinal cognitive and neuroimaging outcomes in the MCSA. Relationships between plasma NfL or T-tau and cognition and neuroimaging in the MCSA stratified by amyloid-beta (Ab) status (elevated Ab PET, A+; non-elevated PET, A−). **A** Associations of baseline plasma NfL or T-tau with cognitive *z*-scores at cross-section. **B** Associations of baseline plasma NfL or T-tau with longitudinal cognitive outcomes. **C** Associations of baseline plasma NfL or T-tau with cross-sectional neuroimaging measures. **D** Associations of baseline plasma NfL or T-tau and longitudinal neuroimaging changes. Models adjust for age, sex, education, and whether or not the cognitive test had been previously administered. WMH volume and infarct measurements were log-transformed. Hippocampal volume measures were adjusted for total intracranial volume. Median follow-up in the cohort was 6.2 years. *Abbreviations: FA* fractional anisotropy, *NfL* neurofilament light, *T-tau* total tau, *WMH* white matter hyperintensity

With regard to cognitive outcomes, higher plasma NfL was cross-sectionally associated with worse global cognitive *z*-scores among those with elevated brain amyloid compared to those without. By contrast, higher plasma T-tau was cross-sectionally associated with worse performance in memory, language, and global cognition (Fig. 2A) for those with elevated brain amyloid compared to those without. Longitudinally, associations between baseline NfL or T-tau and global or domain-specific cognitive decline did not differ by elevated brain amyloid (Fig. 2B).

With regard to neuroimaging outcomes, plasma NfL was cross-sectionally associated with lower corpus callosum FA, but not other outcomes, among those with elevated brain amyloid compared to those without (Fig. 2C). Higher levels of plasma T-tau were cross-sectionally associated with lower cortical thickness in frontal and temporal regions among those with elevated brain amyloid compared to those without (Fig. 2C). Associations between baseline plasma NfL and longitudinal neuroimaging outcomes did not differ by baseline amyloid status (Fig. 2D). In contrast, higher plasma T-tau was associated with greater declines in frontal, and temporal cortical thickness, and a greater increase in WMH volume for those with versus without elevated brain amyloid (Fig. 2D).

### Replication in ADNI cohort

To validate our findings in a separate cohort, we cross-sectionally and longitudinally examined plasma NfL and T-tau in relation to cognitive and neuroimaging outcomes among 387 ADNI participants without dementia. Demographic and clinical characteristics are shown in Table 4 by cognitive status. The group consisted of 190 CU and 197 MCI participants. The median (IQR) age of the cohort was 75.1 (71.6, 79.3), and 61.2% were male. MCI participants had significantly higher median levels of plasma NfL (IQR) (36.9 [27.6, 48.4] vs. 29.2 [22.9, 39.2] pg/mL, *p* < 0.001) than CU, but not T-tau (2.62 [1.77, 3.45] vs. 2.53 [1.78, 3.14], *p* = 0.324). Median follow-up (IQR) for the entire cohort was 3.0 (2.3, 3.2) years.

**Table 4:** ADNI participant baseline characteristics

Characteristic	Data available	CU	Data available	MCI	***p*** value
		Median (IQR) / N (%)		Median (IQR) / N (%)	
Age (years)	190	75.6 (72.5, 78.4)	197	74.9 (70.2, 79.9)	0.296^a^
Male	190	105 (55.3%)	197	132 (67.0%)	0.018^b^
Education (years)	190	16.0 (14.0, 18.0)	197	16 (14, 18)	0.538^a^
≥ 1 APOE ε4	190	50 (26.3%)	197	103 (52.3%)	<0.001^b^
Plasma NfL (pg/mL)	190	29.15 (22.90, 39.15)	197	36.9 (27.600, 48.400)	<0.001^a^
Plasma total tau (pg/mL)	190	2.53 (1.78, 3.14)	197	2.62 (1.770, 3.450)	0.324^a^
Time since baseline (years)	190	3.0 (3.0, 4.0)	197	3.0 (2.0, 3.1)	<0.001^a^
Cognitive test					
MMSE	190	29 (29, 30)	197	27 (25, 28)	<0.001^a^
ADAS-Cog13	190	9.50 (6.00, 12.33)	197	19 (14.670, 23.670)	<0.001^a^
Logical Memory - Immediate Recall	190	13 (12, 16)	197	7 (5, 9)	<0.001^a^
Logical Memory - Delayed Recall	190	12 (10, 15)	197	3 (1, 6)	<0.001^a^
Trail Making Test Part B (sec, max 300)	190	80 (62, 100.75)	197	108 (78, 173)	<0.001^a^
Hippocampal volume (adjusted for TIV)	190	−0.497 (−0.892, −0.097)	197	−1.379 (−1.975, −0.819)	<0.001^a^

^a^Kruskal-Wallis rank sum test

^b^Pearson’s chi-square test

*ADAS-Cog13* Alzheimer’s disease assessment scale-cognitive subscale 13 tasks (higher score = worse performance), *APOE* apolipoprotein E, *IQR* interquartile range, *MCI* mild cognitive impairment, *NfL* neurofilament light

**Fig. 3:**
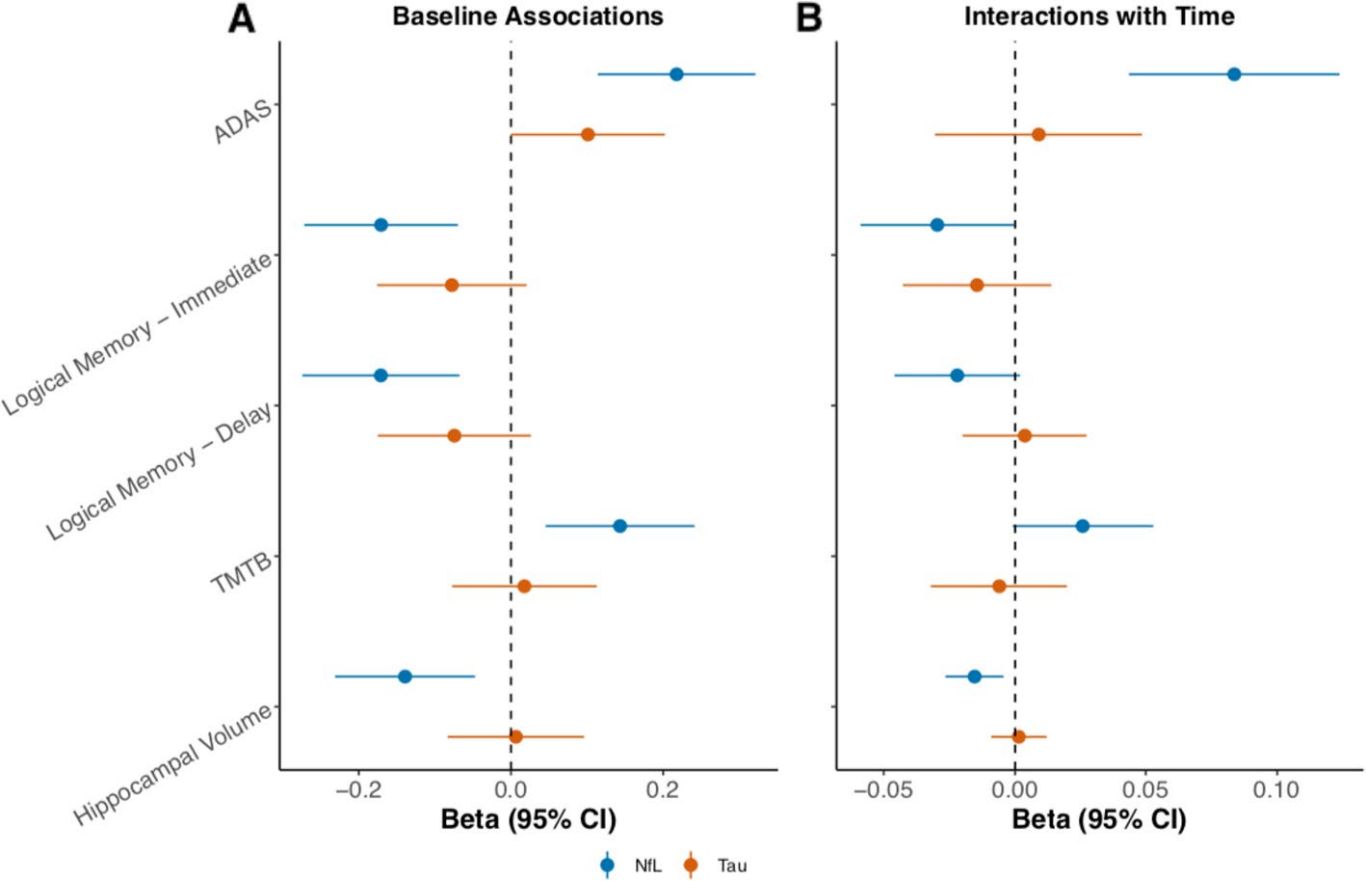
Cross-sectional and longitudinal associations between plasma biomarkers and cognitive and imaging outcomes in ADNI. Relationships between plasma NfL or T-tau and cognition or neuroimaging in ADNI. **A** Associations at cross-section. **B** Associations over time (median follow-up 3.0 years). For the ADAS-Cog13 and Trail Making Test B, a higher score indicates worse performance. Models adjust for age, sex, and education. Hippocampal volume measures were adjusted for total intracranial volume. *Abbreviations: ADAS* Alzheimer’s disease assessment scale-cognitive subscale 13 tasks, *ADNI* Alzheimer’s Disease Neuroimaging Initiative, *TMTB* Trail Making Test Part B

Each one SD increase in baseline plasma NfL was cross-sectionally associated with worse performance on all measures of cognition (Fig. 3A): the ADAS-Cog13, for which a higher score indicates worse performance, Logical Memory – Immediate Recall, Logical Memory – Delayed Recall, and Trail Making Test Part B. Longitudinally, elevated baseline plasma NfL was significantly associated with worse performance on the ADAS-Cog13 and Logical Memory – Immediate Recall (Fig. 3B). T-tau was not cross-sectionally or longitudinally associated with any cognitive outcome. With regard to neuroimaging, elevated baseline plasma NfL was cross-sectionally associated with lower hippocampal volume (Fig. 3A), and with hippocampal atrophy over time (Fig. 3B). There was no association between plasma T-tau and hippocampal volume in cross-sectional or longitudinal analyses.

As with the MCSA analyses, in the ADNI cohort we examined whether the combination of plasma NfL and T-tau in Q4, compared to only one in the top quartile or all in the bottom three quartiles, was more strongly associated with outcomes than either alone (Table 5). Cross-sectionally, Q4 plasma NfL was associated with worse cognitive performance, including a higher ADAS-Cog13, and lower Logical Memory Immediate and Delayed Recall, as well as lower hippocampal volume. Plasma Q4 T-tau was only associated with worse performance on Logical Memory-Delayed Recall. The combination of both plasma NfL and T-tau in Q4, compared to both in the bottom 3 quartiles, was cross-sectionally associated with worse performance on ADAS-Cog13, Logical Memory – Immediate Recall, and Trail Making Test Part B. The combination of both plasma NfL and T-tau in Q4 was also associated with worse performance on the Logical Memory – Delayed Recall, but the strength of the association for the two markers combined was not much different than the associations for either marker alone.

**Table 5:** Cross-sectional and longitudinal associations for Q4 baseline plasma NfL, T-tau, or both in ADNI

Outcome measure	Beta	Lower CI	Upper CI	***p*** value	Beta	Lower CI	Upper CI	***p*** value
	**zNfL Q4**				zNfL Q4 * Time			
ADAS-Cog13	0.400	0.106	0.694	0.008	0.208	0.094	0.321	< 0.001
Logical Memory - Immediate	−0.334	−0.619	−0.048	0.022	−0.083	−0.166	0.000	0.050
Logical Memory - Delayed	−0.370	−0.662	−0.078	0.013	−0.062	−0.132	0.008	0.082
Trail Making Test B	0.189	−0.089	0.466	0.184	0.057	−0.020	0.135	0.148
Hippocampal volume	−0.523	−0.781	−0.265	< 0.001	−0.051	−0.082	−0.021	0.001
	**zT-tau Q4**				zT-tau Q4 * Time			
ADAS-Cog13	0.254	−0.033	0.542	0.084	0.087	−0.027	0.201	0.136
Logical Memory - Immediate	−0.208	−0.487	0.071	0.144	−0.086	−0.168	−0.004	0.042
Logical Memory - Delayed	−0.322	−0.607	−0.037	0.027	0.019	−0.051	0.088	0.598
Trail Making Test B	0.083	−0.188	0.354	0.549	0.003	−0.074	0.080	0.938
Hippocampal volume	−0.061	−0.313	0.191	0.636	−0.001	−0.031	0.030	0.976
	**Q4 Both**				Q4 Both * Time			
ADAS-Cog13	0.526	0.177	0.874	0.003	0.135	−0.002	0.271	0.054
Logical Memory - Immediate	−0.477	−0.816	−0.138	0.006	−0.069	−0.170	0.032	0.183
Logical Memory - Delayed	−0.391	−0.738	−0.045	0.027	−0.079	−0.164	0.006	0.069
Trail Making Test B	0.404	0.075	0.734	0.017	0.015	−0.081	0.111	0.759
Hippocampal volume	−0.028	−0.334	0.278	0.856	−0.010	−0.046	0.027	0.603

For the ADAS-Cog13 and Trail Making Test B, a higher score indicates worse performance. Cognitive measures were *z*-scored prior to analysis except Trail Making Test B, which was *z* log-transformed. Hippocampal volume measures were adjusted for total intracranial volume. Reference level is having both biomarkers in the bottom three quartiles. Models were all adjusted for age, sex, and education

*ADAS-Cog13* Alzheimer’s disease assessment scale-cognitive subscale 13 tasks, *NfL* neurofilament light, *Q4* top quartile, *T-tau* total tau

Longitudinally, the combination of Q4 plasma NfL and T-tau at baseline did not provide prognostic value beyond either marker alone for cognitive decline. Only Q4 NfL alone was longitudinally associated with worse performance on the ADAS-Cog13. Both Q4 NfL and Q4 T-tau were associated with worse performance on Logical Memory – Immediate Recall. However, the coefficients of both markers were very similar and the combination of both markers in Q4 did not add to the prognostic value of either alone. For hippocampal volume, Q4 NfL was associated with lower volume at baseline and decreasing volume over time. There was no association between Q4 T-tau and hippocampal volume.

## Discussion

The emergence of NfL and T-tau in recent years as candidate plasma biomarkers of neurodegeneration merits direct comparison of their relationships with cognition and neuroimaging. It is important to understand the advantage of each plasma neurodegeneration marker for clinical trials endpoints, clinical diagnosis, and prognosis. It is also possible that the utility of the biomarkers will depend on the setting and participant recruitment methodology. For this reason, we examined the relationship between the two plasma neurodegeneration markers and cognitive and imaging outcomes in both the population-based MCSA and in the more clinical setting of ADNI.

In the MCSA, plasma NfL was more strongly associated with cross-sectional and longitudinal global and domain-specific cognitive decline compared to plasma T-tau. However, there was some cross-sectional benefit of including plasma T-tau especially pertaining to the memory domain. The combination of having both NfL and T-tau in the top quartile was more strongly associated with lower memory and global cognitive *z*-scores than either alone. Longitudinally, plasma T-tau did not provide any prognostic value for cognitive decline beyond that provided by plasma NfL. With regard to neuroimaging outcomes, the combination of having both elevated plasma NfL and T-tau, compared to either alone, was more strongly associated with lower temporal lobe thickness and a higher number of infarcts cross-sectionally. Thus, the combination of both markers likely captures overall neurodegeneration at cross-section better than either biomarker alone. Longitudinally, plasma T-tau again did not provide prognostic value for neuroimaging outcomes beyond that provided by plasma NfL. Thus, plasma NfL is a better prognostic marker for neuroimaging outcomes. Results from the MCSA cohort were largely validated using available data from the ADNI cohort, wherein plasma NfL associated cross-sectionally with all cognitive outcomes and longitudinally with worse performance on the ADAS-Cog13 and Logical Memory – Immediate Recall, as well as with cross-sectional and longitudinal hippocampal volume. Plasma T-tau was not cross-sectionally or longitudinally associated with either cognitive or neuroimaging outcomes in the ADNI analysis.

Two recent studies also compared the associations of plasma NfL and T-tau with cross-sectional and longitudinal cognitive outcomes across the AD clinical spectrum, with both concluding that NfL is likely a more useful biomarker for monitoring cognitive status [15, 16]. Although the results presented for both the MCSA and ADNI are largely similar to the previous studies, we did observe that plasma T-tau provided added cross-sectional value to NfL for tests of memory among participants without dementia. Sugarman and colleagues reported that T-tau did not provide added value to NfL for the diagnosis of CU vs. MCI or MCI vs. AD dementia, but the utility of both biomarkers for performance on domain-specific cognitive tests was not examined [16].

While studies have shown that elevated levels of plasma T-tau and NfL are associated with a variety of neuroimaging measures including cortical thickness, cortical atrophy, white matter hyperintensity, or white matter integrity [2–14], the two markers have not been directly compared or assessed for combined additive value. Although we found that plasma NfL was most strongly associated with most imaging measures of vascular pathology, aging-related neurodegeneration, and AD-specific changes, there was again some added cross-sectional value of measuring plasma T-tau. More specifically, participants with both elevated plasma NfL and T-tau, compared to either alone, had significantly lower temporal lobe thickness and a higher number of infarcts at baseline. Longitudinally, the combination of both markers was associated with increasing WMH volume. Thus, measuring both markers may be useful as a way to assess the pathological severity of disease at a given point in time. This value could also extend to cognition since high levels of both markers were more strongly associated with lower global cognitive *z*-scores cross-sectionally than either alone. In contrast, for prognostic purposes, NfL appears to be a better plasma biomarker, with the exception of WMH volume in which the combined information for both markers was better than either alone.

Notably, after stratification by baseline elevated brain amyloid, both cross-sectional and longitudinal associations of plasma NfL with cognition and neuroimaging were largely amyloid-independent. In contrast, some cross-sectional and longitudinal associations of plasma T-tau with cognitive and neuroimaging outcomes were stronger in those with elevated Aβ, including frontal, parietal, and temporal lobe thickness as well as WMH volume. Plasma T-tau may thus be more sensitive to amyloid-dependent changes in the brain.

Future studies examining the pathologic correlates of these biomarkers will also be useful. Plasma NfL is regarded as a nonspecific marker of axonal damage, and may be a better marker of cognitive decline and neuroimaging changes because it is sensitive to multiple pathologies such as AD-related neurodegeneration, cerebrovascular disease, traumatic brain injury, and TDP-43-related pathology [12, 44]. Plasma T-tau may be more limited to cerebrovascular disease and tauopathy-associated neurodegeneration and may thus have constrained utility as a plasma biomarker of all-cause neurodegeneration compared to NfL [8, 45].

### Limitations

A limitation of both the MCSA and ADNI is the lack of racial and ethnic diversity of the samples. Thus, the results may not be generalizable to populations that have a higher prevalence of vascular and other chronic conditions.

## Conclusions

In summary, plasma NfL was a better prognostic marker of cognitive decline and neuroimaging changes in our analyses. Plasma T-tau had little prognostic value but may complement plasma NfL in assessing disease severity at a single timepoint.

## Data Availability

The datasets used and/or analyzed during the current study are available from the corresponding author upon reasonable request: https://www.mayo.edu/research/centers-programs/alzheimers-disease-research-center/research-activities/mayo-clinic-study-aging/for-researchers/data-sharing-resources

## Abbreviations

Aβ: Amyloid-beta
AD: Alzheimer’s disease
ADAS-COG 13: Alzheimer’s Disease Assessment Scale-Cognitive 13
ADNI: Alzheimer’s Disease Neuroimaging Initiative
CDR: Clinical Dementia Rating
CI: Confidence intervals
CU: Cognitively unimpaired
DTI: Diffusion tensor imaging
FA: Fractional anisotropy
IQR: Interquartile range
MCI: Mild cognitive impairment
MCSA: Mayo Clinic Study of Aging
MMSE: Mini-Mental State Examination
MRI: Magnetic resonance imaging
NfL: Neurofilament light chain
Q4: Top quartile
SD: Standard deviation
TIV: Total intracranial volume
T-tau: Total tau
WMH: White matter hyperintensity
